# Statement of Retraction: Baicalin regulates macrophages polarization and alleviates myocardial ischaemia/reperfusion injury via inhibiting JAK/STAT pathway

**DOI:** 10.1080/13880209.2026.2753396

**Published:** 2026-10-28

We, the Editors and Publisher of *Pharmaceutical Biology*, have retracted the following article:

Xu, M., Li, X., & Song, L. (2020). Baicalin regulates macrophages polarization and alleviates ­myocardial ischaemia/reperfusion injury via inhibiting JAK/STAT pathway. *Pharmaceutical Biology*, *58*(1), 655–663. https://doi.org/10.1080/13880209.2020.1779318

Following publication, concerns were raised in 2025 regarding the image integrity of the Western Blot Figures 2D, 4C, 5A, 6B and the qRT-PCR methodology and results described in the article. When approached for an explanation, the authors did not respond to our queries.

As verifying the validity of published work is core to the integrity of the scholarly record, we are therefore retracting the article. The corresponding author has been informed.

We have been informed in our decision-making by our editorial policies and the COPE guidelines.

The retracted article will remain online to maintain the scholarly record, but it will be digitally watermarked on each page as “Retracted”.

